# Semi-supervised active learning using convolutional auto- encoder and contrastive learning

**DOI:** 10.3389/frai.2024.1398844

**Published:** 2024-05-30

**Authors:** Hezi Roda, Amir B. Geva

**Affiliations:** ^1^Electrical and Computer Engineering, Ben-Gurion University, Be'er Sheva, Israel; ^2^InnerEye Ltd CTO, Herzliya, Israel

**Keywords:** active learning, contrastive learning, clustering, semi-supervised learning, human-in-the-loop

## Abstract

Active learning is a field of machine learning that seeks to find the most efficient labels to annotate with a given budget, particularly in cases where obtaining labeled data is expensive or infeasible. This is becoming increasingly important with the growing success of learning-based methods, which often require large amounts of labeled data. Computer vision is one area where active learning has shown promise in tasks such as image classification, semantic segmentation, and object detection. In this research, we propose a pool-based semi-supervised active learning method for image classification that takes advantage of both labeled and unlabeled data. Many active learning approaches do not utilize unlabeled data, but we believe that incorporating these data can improve performance. To address this issue, our method involves several steps. First, we cluster the latent space of a pre-trained convolutional autoencoder. Then, we use a proposed clustering contrastive loss to strengthen the latent space's clustering while using a small amount of labeled data. Finally, we query the samples with the highest uncertainty to annotate with an oracle. We repeat this process until the end of the given budget. Our method is effective when the number of annotated samples is small, and we have validated its effectiveness through experiments on benchmark datasets. Our empirical results demonstrate the power of our method for image classification tasks in accuracy terms.

## 1 Introduction

In recent years, computer vision has made significant advancements, primarily driven by machine learning and, more specifically, deep learning. However, these methodologies are highly dependent on having a substantial number of labeled samples. Acquiring such a large volume of data poses a significant challenge for several reasons. Initially, the process of annotating images is time-intensive, ranging from a few seconds for simple image classification to several hours for more complex image segmentation tasks. This makes it impractical to annotate a large data set in a short time frame. Additionally, image annotation often requires specialized expertise, adding another layer of complexity. In some cases, annotations require professionals, which increases the cost and complexity of the annotation process.

An effective strategy to address these issues involves employing an active learning methodology. Active Learning, often abbreviated as AL, entails the process of selecting and prioritizing data that require labeling to have the most significant impact on the training of a machine learning task. Through the utilization of AL, machine learning algorithms can enhance their accuracy using a reduced number of training labels, thereby economizing time and resources during model training. Settles (2009) provides a comprehensive overview of various AL techniques in machine learning. In essence, there are three primary scenarios where active learning can be beneficial for those seeking to maximize accuracy while minimizing the number of labeled instances, typically involving the submission of queries in the form of unlabeled data instances to be labeled by an oracle, such as a human annotator. These scenarios include membership query synthesis (Angluin, 1988), stream-based selective (Atlas et al., 1989) sampling, and pool-based sampling. In this research, we will be focused on the third scenario, pool-based sampling (Lewis, 1995).

In numerous practical scenarios, it is often straightforward to gather a substantial amount of unlabeled data, which serves as a driving force behind the adoption of the pool-based sampling method. Let us consider a pool of unlabeled data *P*_*u*_ alongside a limited quantity of labeled data *P*_*l*_. In pool-based sampling in each query, we will sample a small amount of data from *P*_*u*_ and annotate it with human oracle, then add it to *P*_*l*_. Assuming we have a good query that selects the most relevant samples from *P*_*u*_, *P*_*l*_ will be a good representative group of *P*_*u*_.

Employing a pool-based sampling active learning approach, where the model selects samples for annotation, can decrease the quantity of labeled data required to achieve a similar model accuracy. This represents a significant benefit of active learning for deep learning tasks, which has only recently started to be investigated (Gal et al., 2017; Sener and Savarese, 2017; Sinha et al., 2019).

As previously mentioned in numerous practical scenarios, there is a significant volume of unlabeled data, which motivate our study. In this research, we present a novel approach that utilizes pool-based active learning to fully exploit all unlabeled data. The method we suggest begins by clustering the unlabeled data in the latent space. Then, it proceeds to choose the samples with the highest entropy based on their representation in the latent space and the clustering within that space. Our central concept involves clustering the unlabeled data from *P*_*u*_, querying samples with the highest entropy for human annotation, and employing labeled data from *P*_*l*_ to refine the clustering via our suggested clustering contrastive learning. The above process iterates until either a satisfactory level of accuracy is achieved, the model converges, or the annotation budget is exhausted.

In addition to addressing the challenges posed by limited labeled data, our research holds promise for real-world applications where unlabeled data is abundant. By leveraging a pool-based active learning approach, our method enables the effective utilization of unlabeled data in scenarios where acquiring labeled samples is impractical or costly, such as medical imaging diagnosis, satellite image analysis, and industrial inspection. This capability maximizes the efficiency and effectiveness of machine learning models in practical settings, facilitating improved accuracy and insights from limited labeled samples. Furthermore, our approach can identify and prioritize hard examples for labeling, ensuring that the annotated data provide the most informative training signal for the model.

The contributions of the research are:

- A new approach is proposed to integrate Deep Clustering and Deep Active Learning (DAL) in order to maximize the extraction of information from both labeled and unlabeled data.
- Propose a novel *contrastive clustering loss* (CCL) that has the potential to enhance the transition from unsupervised clustering to a semi-supervised framework.
- Achieving a high level of accuracy in image classification with a reduced number of labeled samples.

## 2 Previous work

### 2.1 Deep clustering

There has been significant research on deep clustering in recent years. Most deep clustering algorithms can be categorized into two groups. The first group includes two-stage clustering algorithms that first generate a data representation before applying clustering. These algorithms leverage existing unsupervised deep learning frameworks and techniques. For instance, Tian et al. (2014) and Peng et al. (2016) utilize autoencoders to learn low-dimensional features of original data samples and subsequently apply conventional clustering algorithms like k-means to the learned representations. Mukherjee et al. (2019) introduces ClusterGAN a generative adversarial network that clusters the latent space by sampling latent variables from a combination of one-hot encoded variables and continuous latent variables. The second group comprises approaches that simultaneously optimize feature learning and clustering. These algorithms aim to explicitly define a clustering loss, resembling the classification error in supervised deep learning. Yang et al. (2016) propose a recurrent framework that integrates feature learning and clustering into a unified model with a weighted triplet loss, optimizing it end-to-end. Xie et al. (2016) suggests a clustering loss that operates on the latent space of an autoencoder, enabling the simultaneous acquisition of feature representations and cluster assignments. Building upon this, Guo et al. (2017) DCEC (Deep Clustering with Convolutional Autoencoders) enhances the method by proposing Convolutional Autoencoders (CAE), which surpasses DEC while ensuring the preservation of local structure. This study directly adopts the clustering loss and clustering layer from DCEC.

We briefly review their definitions:

The trainable parameters of the clustering layer are ${\mu_{j}}_{1}^{k}$ which represent the cluster center. The intuition behind the math operation of that layer is it maps each embedded point in the latent space *z*_*i*_ into a soft label *q*_*i*_ by the student's t-distribution (Van der Maaten and Hinton, 2008).


(1) $$q_{ij}=\frac{{\left(1+||z_{i}-\mu_{j}|{|}^{2}\right)}^{-1}}{\sum_{j}{\left(1+||z_{i}-\mu_{j}|{|}^{2}\right)}^{-1}}$$

Where *q*_*i*_*j* is the *j*th entry of *q*_*i*_, representing the probability of *z*_*i*_ belonging to cluster *j*.

The clustering loss is defined as:


(2) $$\begin{array}{l} L_{clu}=KL\left(P||Q\right)=\underset{i}{\sum }\underset{j}{\sum }p_{ij}\log\frac{p_{ij}}{q_{ij}} \end{array}$$

where *P* is the target distribution, defined as:


(3) $$p_{ij}=\frac{q_{ij}^{2}/\sum_{i}qij}{\sum_{j}\left(q_{ij}^{2}/\sum_{i}qij\right)}$$

### 2.2 Active learning

Active learning is a subfield of machine learning empowering algorithms to select and prioritize the most informative data points for labeling, aiming to enhance model performance using less training data. Active learning scenarios commonly occur in three main contexts:

1. Membership Query Synthesis: In this scenario (Angluin, 1988), the learner synthesizes new instances to be labeled by an oracle, aiming to generate maximally informative instances, particularly beneficial when labeled data is scarce or expensive to obtain. 2. Stream-Based Selective Sampling: This scenario (Atlas et al., 1989) involves a continuous stream of unlabeled instances, with the learner making real-time decisions on which instances to label based on the current model state and incoming data. Such scenarios are common in sequential data streams like online learning or sensor data. 3. Pool-Based Sampling: Here (Lewis and Gale, 1994), the learner is presented with a fixed pool of unlabeled instances and selects a subset for labeling, aiming to identify the most informative instances. This approach involves evaluating the informativeness of unlabeled samples, often utilizing query strategies like uncertainty sampling (Lewis and Gale, 1994), recently Liu and Li (2023) had an extensive work to explain this strategy even further, or query-by-committee (Seung et al., 1992). Active learning plays a crucial role in determining which data should be labeled to maximize the effectiveness of training supervised models. Traditional active learning methods are comprehensively reviewed by Settles (2009), while Ren et al. (2021) offer insights into the more contemporary Deep Active Learning (DAL) approach, integrating active learning with deep learning methodologies.

Notable active learning methodologies are Uncertainty Sampling (Lewis and Gale, 1994) and Variational Adversarial Active Learning (VAAL) (Sinha et al., 2019). VAAL integrates variational inference and adversarial training, leveraging a generator network to produce informative data points and a discriminator network to differentiate between real and generated instances, aiding in sample selection. Additionally, LADA (Kim et al., 2021) introduces data augmentation techniques to improve the efficiency of data acquisition in deep active learning, while SRAAL (Zhang et al., 2020) integrates adversarial training techniques with active learning principles to address sample selection challenges.

Moreover, approaches like the Core-Set Approach (Sener and Savarese, 2017) and Bayesian Active Learning (BALD) (Houlsby et al., 2011) offer strategies for selecting informative instances, with Core-Set identifying a compact, diverse subset of unlabeled data, and BALD leveraging Bayesian inference for strategic instance selection. These methodologies collectively contribute to enhancing model training efficiency and performance in active learning settings.

### 2.3 Semi-supervised learning

Semi-supervised learning (SSL) is a specialized form of supervised learning that involves training on a small set of labeled data along with a large set of unlabeled data. Positioned between supervised and unsupervised learning, SSL is commonly used in scenarios where the availability of labeled data is limited due to constraints such as budgetary restrictions or data ambiguity, where the class of a sample is uncertain. Semi-supervised algorithms are designed to address such challenges. In this study, we propose an SSL approach for the classification of image data, aiming to leverage the benefits of both active learning (AL) and SSL. To achieve this, we suggested clustering contrastive loss (CCL) in conjunction with unsupervised training.

### 2.4 Entropy

Entropy Shannon (1948) is an information-theoretic measure of uncertainty. It quantifies the amount of information needed to encode a distribution. In active learning, entropy is widely used to select the most uncertain or ambiguous samples for annotation. The entropy can be shown as:


(4) $$\begin{array}{l} H\left(x\right)\;:=-\underset{x\in X}{\sum }p\left(x\right)\log p\left(x\right) \end{array}$$

## 3 Method

This study proposes a novel active learning approach based on pool-based sampling. It involves training a convolutional autoencoder (CAE) (Masci et al., 2011) to learn a low-dimensional latent space for both labeled and unlabeled samples. The latent space is then clustered using a clustering layer. After each iteration of the active learning process, a subset of data points associated with the latent space vectors is selected for annotation. To leverage information from the labeled data, the study introduces the contrastive clustering loss (CCL), which is a modified version of the contrastive loss (Chopra et al., 2005). The CCL operates on the latent space vectors, pulling samples of the same class toward their respective cluster centers and pushing samples of different classes apart.

### 3.1 Problem definition and notation

The main focus of this study is a semi-supervised active learning approach designed for image classification. Assuming there is a large set of unlabeled images *P*_*u*_ and a small set of labeled images *P*_*l*_, along with a predetermined annotation budget, the goal is to select the most informative samples from the unlabeled set *P*_*u*_ to enhance the classification accuracy. These selected samples will be labeled by a human annotator and incorporated into the labeled set *P*_*l*_. The initial step involves training a Convolutional Autoencoder (CAE) to learn a condensed representation of the images, referred to as latent space features. Each image *i* is transformed by the CAE into a feature vector *z*_*i*_ in the latent space. Subsequently, all latent space features *z*_*i*_, ∀*i*∈*P*_*l*_∪*P*_*u*_ are clustered into clusters, denoted as μ_*j*_ where *j* represents the centroid of the *j*−*th* cluster. Finally, the proposed cluster contrastive loss *L*_*ccl*_ (see Eq. 7) is applied to the labeled samples *z*_*l*_, ∀*l*∈*P*_*l*_. This loss function aims to attract the feature vectors *z*_*j*_ toward μ_*j*_ while pushing them away from μ_*n*_ ∀*n*≠*j*. for all *n*≠*j*.

### 3.2 Suggested method

The primary objective of this study is image classification, aiming to categorize images into their respective classes with optimal accuracy by leveraging labeled images from the restricted labeled data pool *P*_*l*_. To achieve this, we introduce a pool-based active learning strategy that integrates contrastive learning and clustering, mutually enhancing their performance in every training cycle. Our approach follows a human-in-the-loop methodology, in which an active learning loop comprises model training, image quering, and annotation by an oracle. This iterative process continues until the budget is fully utilized.

**Figure 1:**
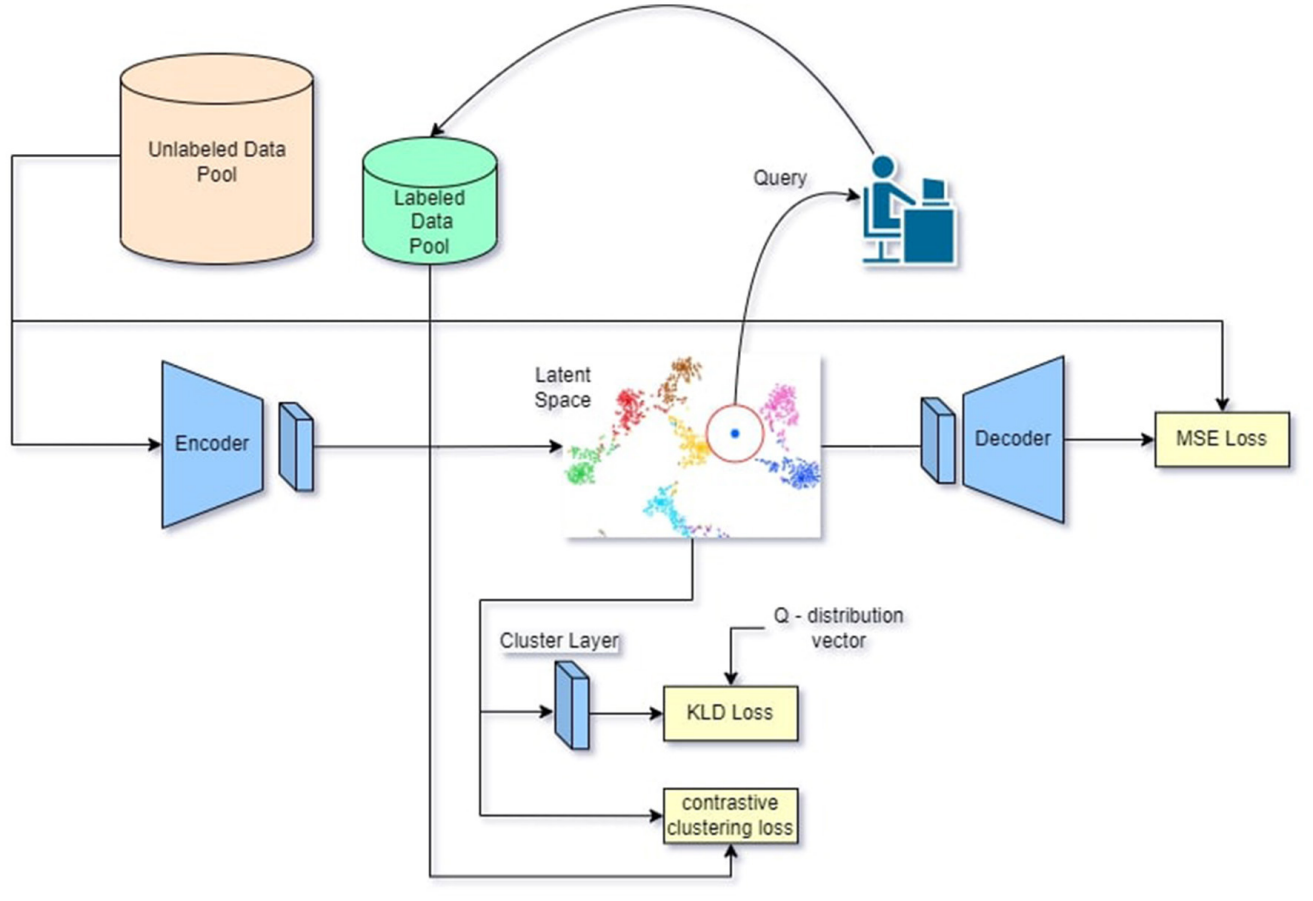
Visual representation of proposed methodology. Images from *p*_*l*_ and *p*_*u*_ are inferred through the CAE and provide feature vectors in the latent space the feature vectors are clustered by the clustering layer and the contrastive clustering loss then the n-th feature vectors from the latent space with the highest entropy are queried and annotated by a human oracle this process is repeated until the end of the annotation budget or model convergance.

The model consists of a CAE (Masci et al., 2011) and a clustering layer (Xie et al., 2016). Samples from *P*_*l*_ and *P*_*u*_ are fed into the model based on the active learning training stage. During each iteration of the active learning process, samples from *P*_*u*_ are chosen for labeling. The proposed module is depicted in Figure 1.

Prior to commencing the active learning iteration, certain initial steps are carried out. Initially, our CAE is pre-trained by reconstructing images from *P*_*u*_ and *P*_*l*_ using the MSE loss (Eq. 5). This process allows the CAE to acquire knowledge of lower-dimensional features within the dataset. Once the network is trained, the resulting latent space provides a feature *z*_*i*_∀*i*∈*p*_*i*_∪*p*_*l*_. Subsequently, the cluster centroids in the clustering layer are initialized with the average values of the vectors in the latent space of each class in our labeled pool *P*_*l*_ as depicted in Eq. 6.


(5) $$\begin{array}{l} L_{rec}=\frac{1}{n}\overset{n}{\underset{i=1}{\sum }}{\left(Y_{i}-\hat{Y_{i}}\right)}^{2} \end{array}$$

(6) $$\begin{array}{l} \mu_{c}=\frac{1}{n_{c}}\overset{n_{c}}{\underset{1}{\sum }}z_{c} \end{array}$$

Next, we incorporate clustering into the training of the CAE by clustering the acquired latent space with the utilization of a clustering layer (Guo et al., 2017) and employing a Kullback-Leibler divergence loss (Csiszár, 1975) as shown in Eq. 2. The primary objective of this stage is to organize the latent space into clusters, ensuring that similar image pairs produce proximate feature vectors within the latent space.

In the final stage, we incorporate the image labels from *P*_*l*_. To utilize these labels effectively, we employ the suggested cluster contrastive loss *L*_*ccl*_ as shown in Eq. 7 on all vectors in the latent space derived from *P*_*l*_, meaning that solely annotated images are taken into account by this loss. The CCL loss works by either pulling or pushing the feature vectors *Z*_*i*_ in the latent space toward their respective cluster center μ_*i*_, or away from other cluster centers μ_*j*_ where *j*≠*i*. This method allows us to enhance the purity of clusters while using a limited number of labeled images from *P*_*l*_, during this stage we continue to make use of the previous clustering stage. Finally, we add all those losses and update the parameters of the model. The process is reiterated until reaching convergence or utilizing the entire annotation budget.

At the end of every active learning iteration, we perform query sampling to choose the n-th image that exhibits features with the highest entropy compared to all other clusters. These features are the most ambiguous in terms of their cluster assignment, and by labeling them, we gain valuable insights that the model failed to generalize. Algorithm 1 presents a generic pseudo-code for this approach, in Table 1 the symbols used in the algorithm are elucidated, providing clarity on their respective meanings and roles within the context of the algorithm.

**Algorithm 1:**
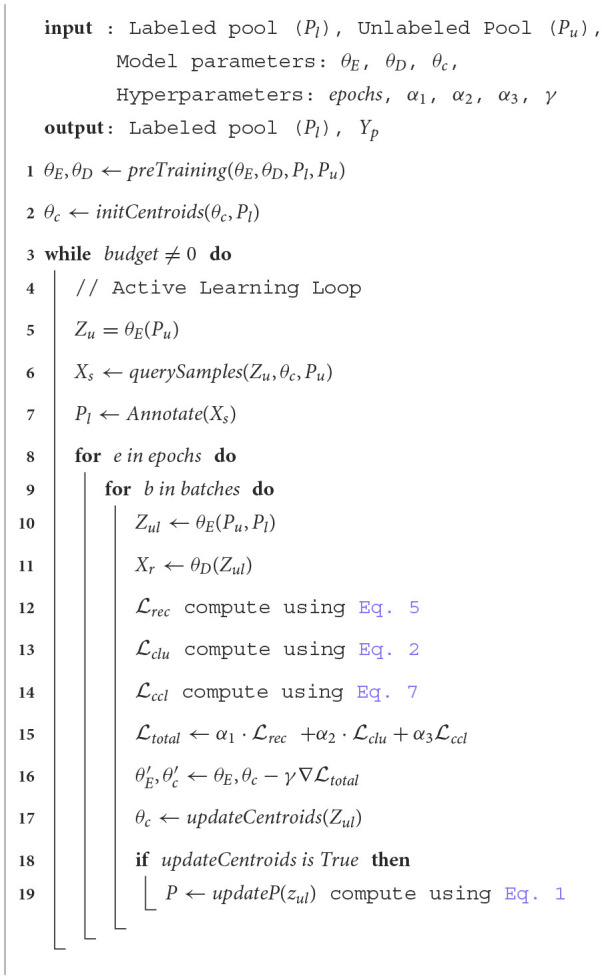
Contrastive active learning.

**Table 1:** Algorithm symbols and their explanations.

**Notation**	**Explanation**
*P*_*l*_	Labeled pool
*P*_*u*_	Unlabeled pool
θ_*E*_	Encoder model parameters
θ_*D*_	Decoder model parameters
θ_*c*_	Centroid parameters
α_1_, α_2_, α_3_	Losses weights
γ	Learning rate
*Z*_*u*_	Encoded representations of unlabeled pool
*X*_*s*_	Samples selected for annotation
*P*_*l*_	Updated labeled pool
*Z*_*ul*_	Encoded representations of both labeled and unlabeled pool
*X*_*r*_	Reconstructed samples
$\theta_{E}^{\prime }$, $\theta_{c}^{\prime }$	Updated encoder and centroid parameters
∇	Gradient operator
*P*	P distribution

#### 3.2.1 Cluster contrastive loss

The cluster contrastive loss (CCL) is a revised variant of the supervised contrastive loss introduced in Khosla et al. (2020). To enhance the purity of the clusters, the proposed approach incorporates the labeled images from *P*_*l*_ into the clustering procedure. Consequently, this results in the adoption of the proposed CCL. The mathematical expression for the CCL is displayed below:


(7) $$\begin{array}{l} L_{ccl}=-\underset{c\in C}{\sum }\underset{i\in I_{c}}{\sum }\log\frac{exp\left(z_{i}\cdot \mu_{c}/\tau \right)}{\underset{z^{\prime }\in I_{c^{\prime }}}{\sum }exp\left(z^{\prime }\cdot \mu_{c}/\tau \right)} \end{array}$$

**Figure 2:**
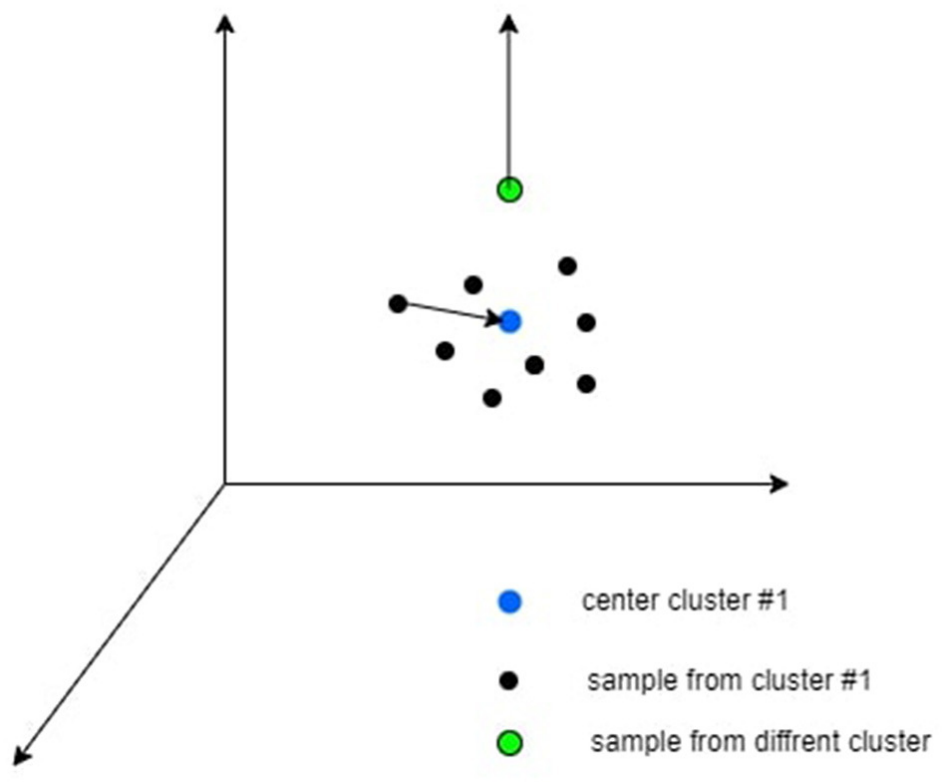
An intuitive explanation of the contrastive clustering loss is that the black dots correspond to samples assigned to cluster #1, the blue dot symbolizes the cluster center, and the green dot represents a sample from a different cluster. This loss function aims to move the black dots closer to the blue dot while pushing the green dot farther away from the blue dot.

Where *c*∈*C* is the class index, *I*_*c*_ is the set of all the samples indexes in class c, $I_{c^{\prime }}$ is the set of all the samples indexes in all the classes beside class c. *z*_*i*_ is the i-th sample in the latent space and μ is the center of the cluster, τ∈*R*^+^ is a scalar temperature parameter. An intuition of the loss can be shown in Figure 2.

This loss involves both pulling samples toward their cluster center and pushing from other unmatched centroids centers simultaneously. It specifically affects the labeled data points. The CCL serves as a complementary approach to the unsupervised methods we currently employ, and empirical experiments indicate their mutual benefit. Figure 2 provides a visual representation of CCL as defined in Eq. 7.

#### 3.2.2 The need for the contrastive clustering loss

During the training for CAE, we are provided with representation vectors in the latent space. In order to group the latent space into clusters corresponding to each class, as elaborated in Section 2.1, the clustering layer is utilized. This layer aims to streamline the process of image classification. Nevertheless, the clustering mechanism is proficient in grouping vectors with high certainty, which may result in certain images not being grouped together, particularly those from the same class that map to distant vectors in the latent space. Therefore, the integration of the suggested contrastive clustering loss becomes essential. This suggested CCL loss function works on adjusting vectors that were not properly aligned by the clustering process. Through this loss function, we can enhance the separation of classes in the latent space, even when dealing with a limited number of labeled images or when images are challenging to cluster due to the low confidence in the P-distribution of the clustering process.

#### 3.2.3 Pre-training

During the initial phase, we train the convolutional autoencoder. We are using all the images from the unlabeled data pool *P*_*u*_ and the labeled data pool *P*_*l*_. Each image *x*_*i*_~*P*_*l*_∪*P*_*u*_ inferences through the encoder and provides *z*_*i*_ a lower dimension latent vector *z*_*i*_ = σ(*x*_i_*W)) where *w* is the weights of the encoder layers, σ is a nonlinear activation function, and * is a convolution operation. The latent vector *z*_*i*_ is inference through the decoder which provides an $\hat{x}$ which is a reconstruction of the original image *x*_*i*_. $\hat{x}=\sigma \left(z_{i}*U\right)$ where *U* is the weight for the decoder. $\hat{x_{i}}$ and *x*_*i*_ are entered to MSE loss (Eq. 5) which provides a high loss when *x*_*i*_ looks different from $\hat{x_{i}}$ and a low loss when they are similar. At the end of this step, the CAE has trained weights *W* and *U*.

#### 3.2.4 Initialization and update centroids

Once the CNN is pre-trained, the centroids in the clustering layer are initialized using the average value of each class projection from *P*_*l*_ in the latent space. Subsequently, every 80 iterations, the distribution of *P* is updated by the following (Eq. 3). As detailed in Section 2.1, the centroids represent the weights of the clustering layer, and therefore they are adjusted during each training iteration.

#### 3.2.5 Query samples

**Figure 3:**
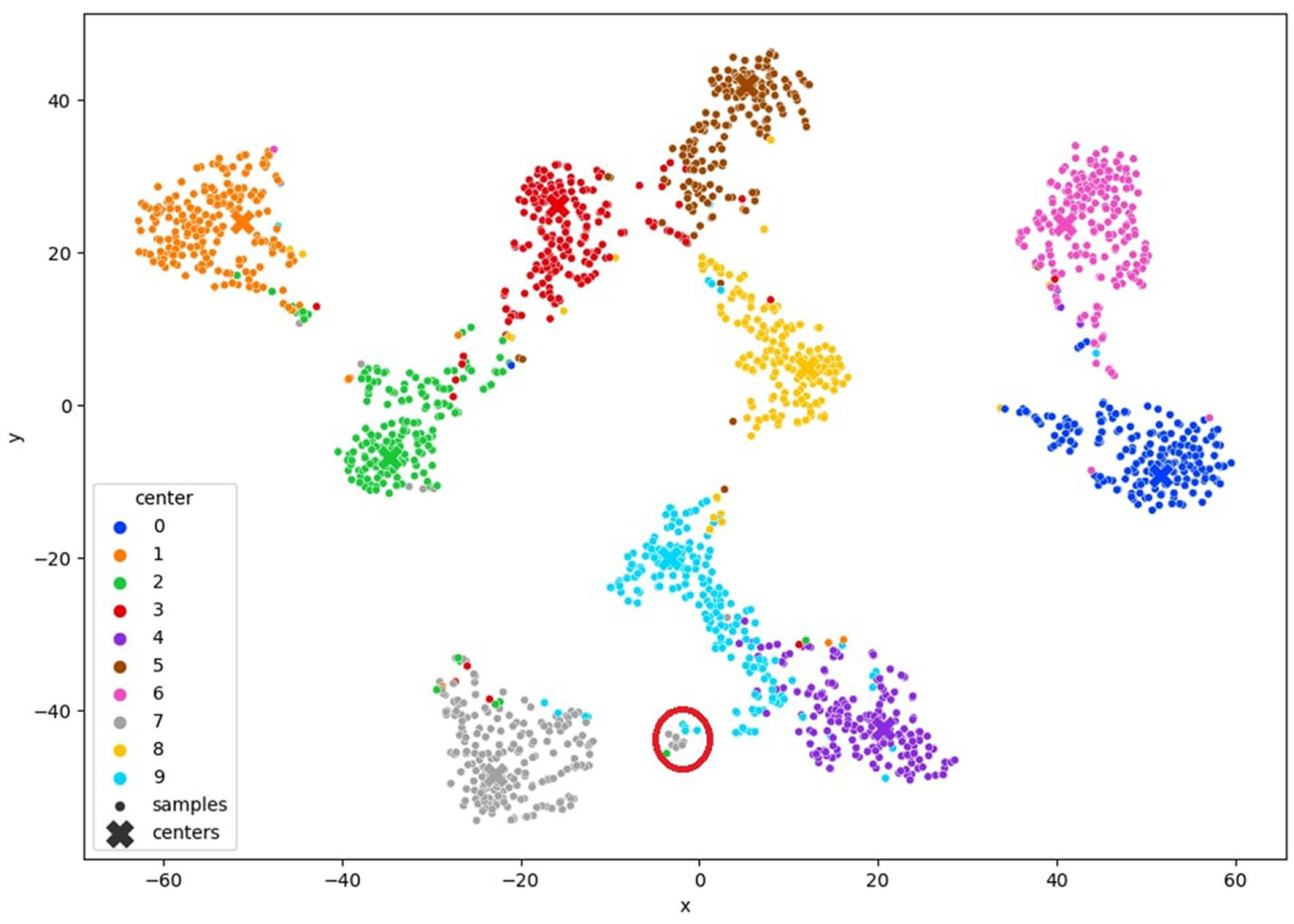
TSNE visualization of the query method the red circle represents samples with high entropy.

In this stage, our objective is to acquire image annotations by engaging a human annotator in the active learning procedure. At this point, we have already acquired a clustered latent space generated by the model itself. Any vectors within the latent space that are not clustered or are distant from the cluster center are identified as hard examples, representing images that require annotation. We select samples linked to vectors in the latent space that do not clearly belong to any cluster and annotate them based on the uncertainty criterion detailed in Eq. 4. More specifically, we target the vectors that exhibit the highest entropy in the cluster distribution. A visual representation of this approach is shown in Figure 3. By focusing on a small number of samples associated with feature vectors located far from the cluster center, we gain insight into these samples and the clusters they are associated with, thereby enhancing the overall clustering process.

### 3.3 Combination of contrastive learning and clustering

When the suggested clustering method is applied to the latent space, there may be instances where some feature vectors are not accurately clustered. This situation can arise when feature vectors within the latent space that should belong to the same cluster are spatially distant from each other. As a result, the clustering layer may encounter challenges in grouping these feature vectors effectively. To address this issue, we introduce our proposed CCL, which works to minimize the distance between distant feature vectors that belong to the same cluster while maximizing the separation between those that do not. Furthermore, we incorporate a query mechanism to select challenging examples (i.e., samples that are significantly distant from their corresponding cluster center) for manual annotation. By integrating these strategies and progressively bringing the feature vectors closer together in a semi-supervised fashion, followed by clustering using the clustering layer, we improve the purity of the clustering outcomes.

### 3.4 Implementation details

In this work, we used a convolutional autoencoder for our model. The encoder consists of 3 convolutional layers, a batch normalization layer, and a linear embedding layer with a size of 10. The decoder consists of a linear de-embedding layer, 3 deconvolutional layers, and a batch normalization layer. The clustering layer weights are initialized with the mean of the latent space clusters using the starting labeled images in *P*_*l*_, and are then updated with the kl-loss using the Q and P distribution as described earlier. The P-distribution, or target distribution, is initialized every 80 steps. Each benchmark dataset is split into a 20% validation set and 80% training set, which is further divided into two data pools: a labeled data pool *P*_*l*_ and an unlabeled data pool *P*_*u*_. First, we pre-trained the model for 50 epochs. Then each active learning training iteration was set to 10 epochs and for the duration of overall 20 active learning loops. In each active learning loop, we query 250 image samples using the uncertainty strategy for annotation.

## 4 Experiments and results

### 4.1 Datasets

**Figure 4:**
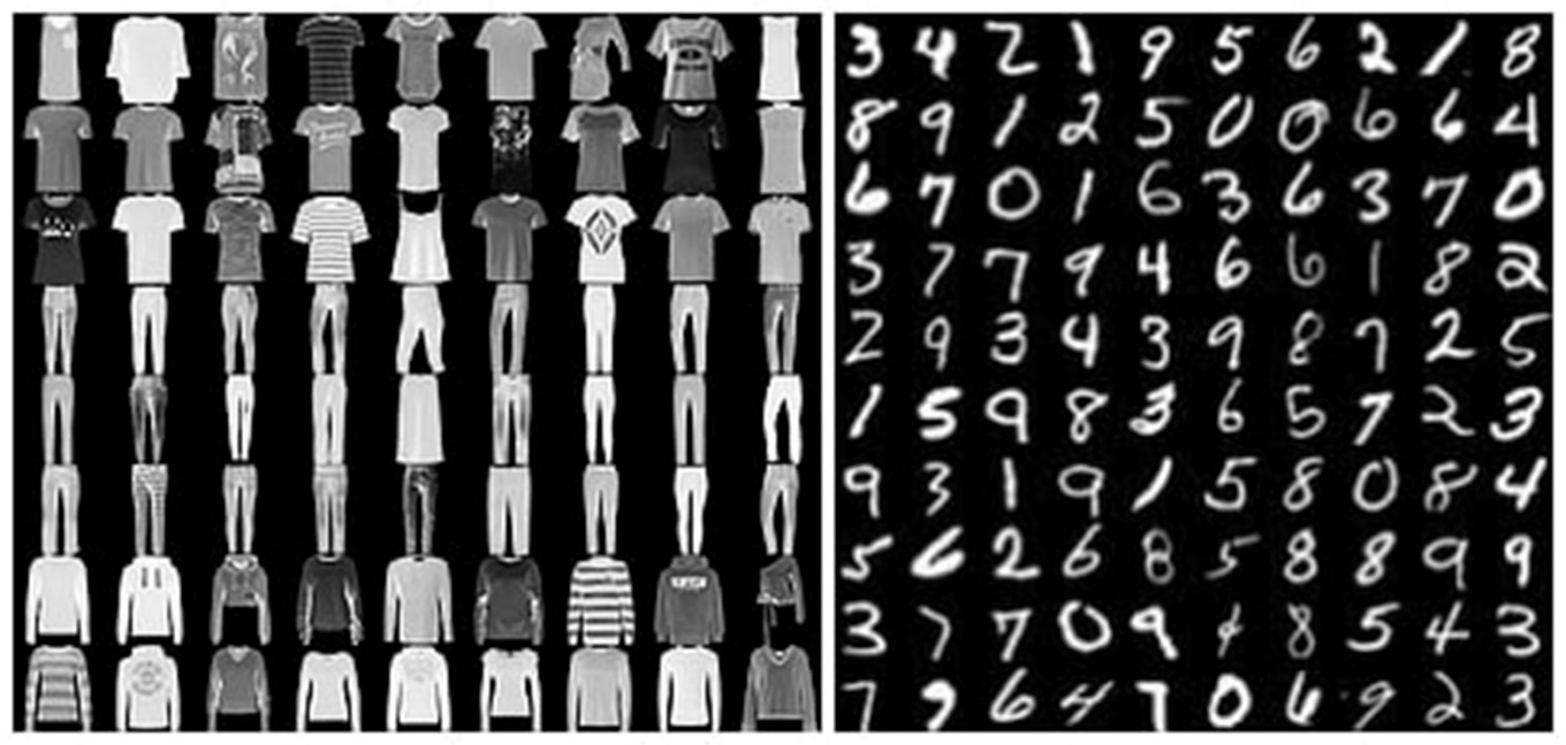
Visualization of MNIST and FashionMNIST datasets at the **left** is the FashionMNIST and on the **right** is the MNIST dataset.

**Figure 5:**
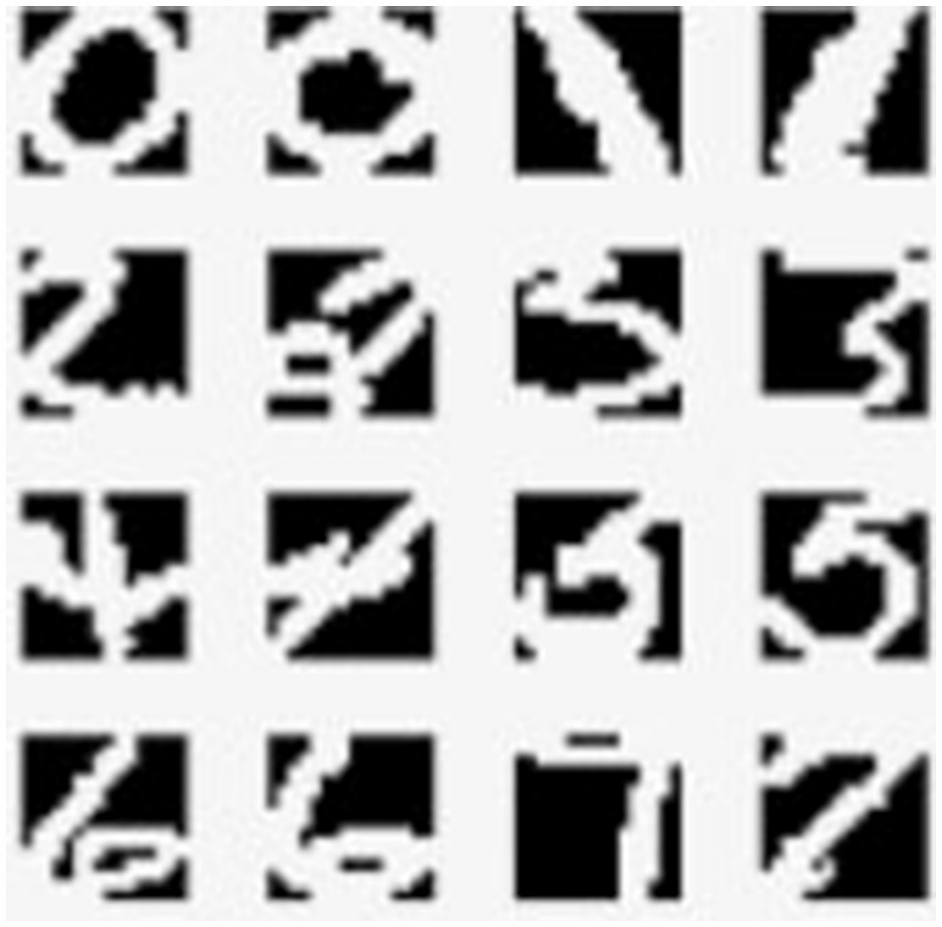
Visualization of the USPS dataset.

We have evaluated our method in image classification tasks. We have used MNIST (LeCun, 1998), FashionMNIST (Xiao et al., 2017), and USPS (Hull, 1994) datasets. Both the MNIST and the FashionMNIST datasets have 60K grayscale images of size 28x28. Examples of MNIST and FashionMNIST datasets can be viewed at Figure 4, and USPS has 9298 grayscale images of 16x16 size. An example of USPS dataset can be viewed at Figure 5.

### 4.2 Performance measurement

We evaluate the performance of our method with the image classification task by measuring the accuracy over different amounts of labeled images from 500 to 5k images with a raising of 250 images from query to query. The results of all our experiments are averaged over 3 runs.

### 4.3 Experiments details

We begin our experiments with an initial labeled pool of the size of 250 and in each iteration of the training loop we provided another 250 images that were annotated by the human oracle and added to the initial labeled pool *P*_*l*_. Training is repeated on the new training set with the new labeled images. We assume that the dataset is balanced and the oracle annotations are ideal.

**Figure 6:**
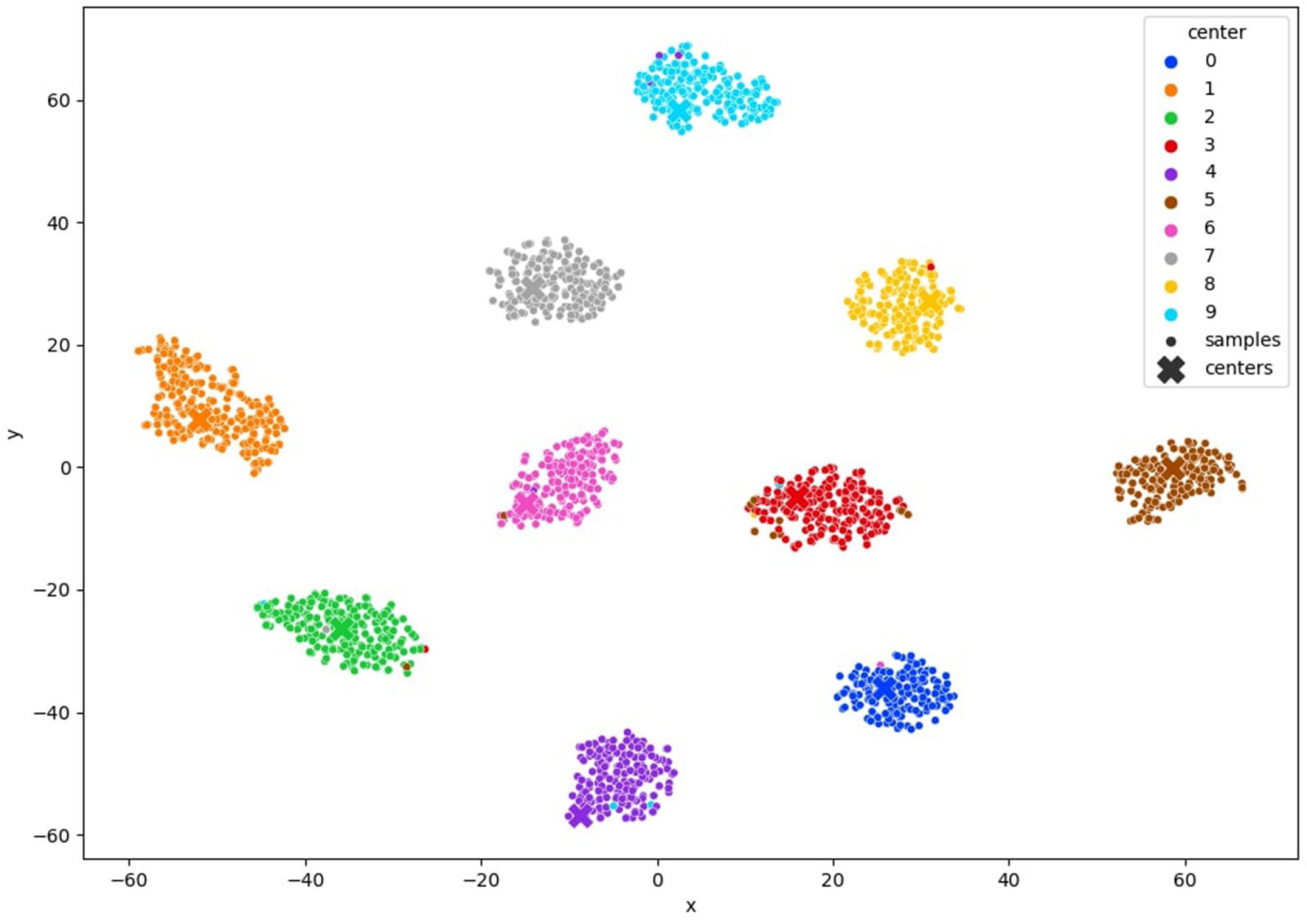
TSNE visualization of the clustered MNIST latent space after convergence of our method with 10% of annotated samples.

**Figure 7:**
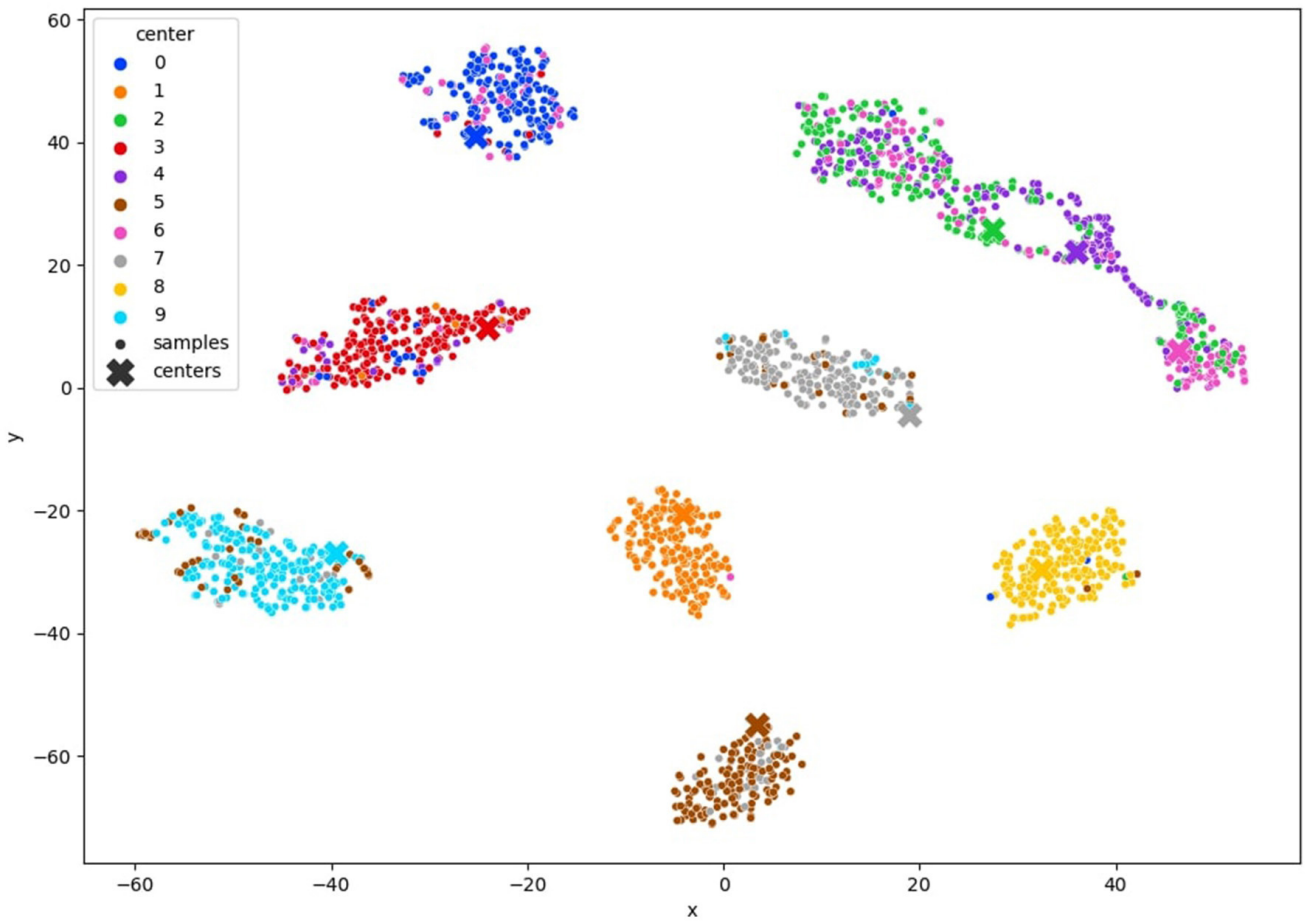
TSNE visualization of the clustered FashionMNIST latent space after convergence of our method with 10% of annotated samples.

In Figure 6 MNIST result. In Figure 7 FashionMNIST result.

### 4.4 Effectiveness of the CCL

**Figure 8:**
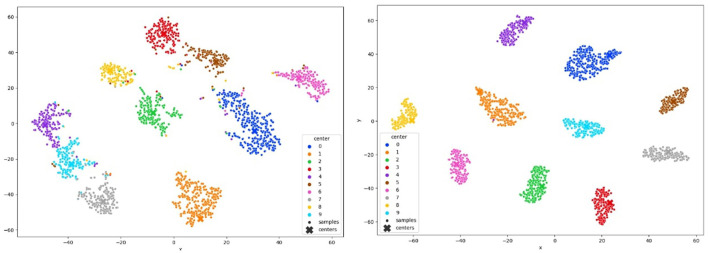
**On the left**: t-SNE visualization after clustering. **On the right**: t-SNE visualization after applying CCL in conjunction with clustering.

In Table 2, we present an ablation study comparing our proposed method with the use of clustering alone. The study evaluates the performance of both approaches on the Mnist and USPS datasets. The results demonstrate that integrating the CCL with clustering, using only 3% of labeled data, significantly improves model performance. The CCL operates by encouraging the model to learn discriminative representations within clusters while simultaneously enforcing compactness among cluster centroids. By incorporating this loss function into our framework, we guide the clustering process to yield clusters that not only capture inherent data structures but also ensure inter-class separability. This results in more coherent and well-separated clusters, facilitating better decision boundaries and ultimately leading to improved classification accuracy. Additionally, Figure 8 visually illustrates the difference between using clustering alone and incorporating the CCL into the clustering process.

**Table 2:** Ablation study: clustering vs. clustering + CCL (3% of annotated data).

**Dataset**	**Method**	
	**Clustering**	**Clustering + CCL**
MNIST	81.6%	91.0%
USPS	68.7%	86.5%

### 4.5 Comparing with other methods

**Figure 9:**
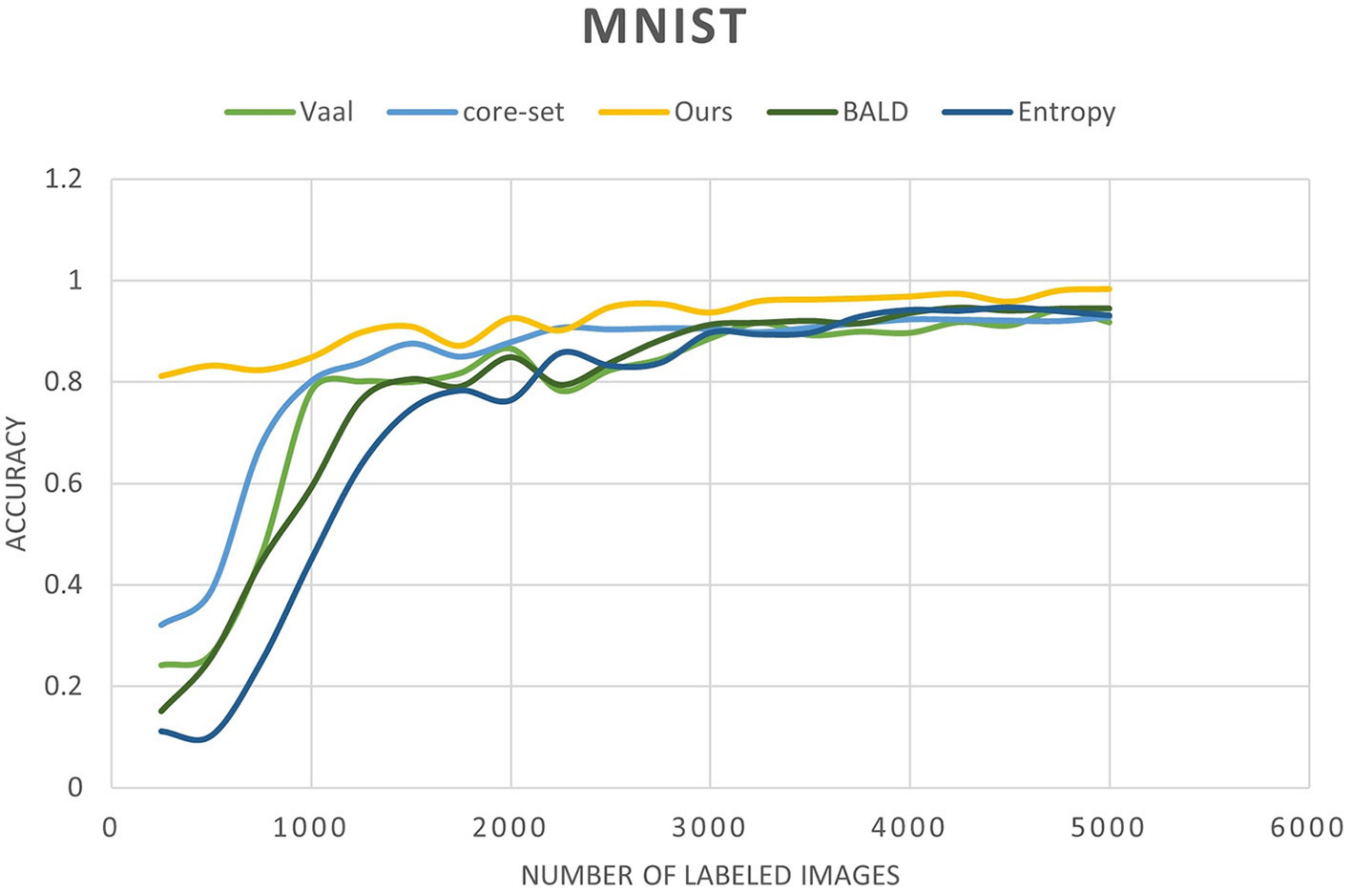
Accuracy of our method compared to other state-of-the-art methods as a function of the number of labeled images for the MNIST dataset.

**Figure 10:**
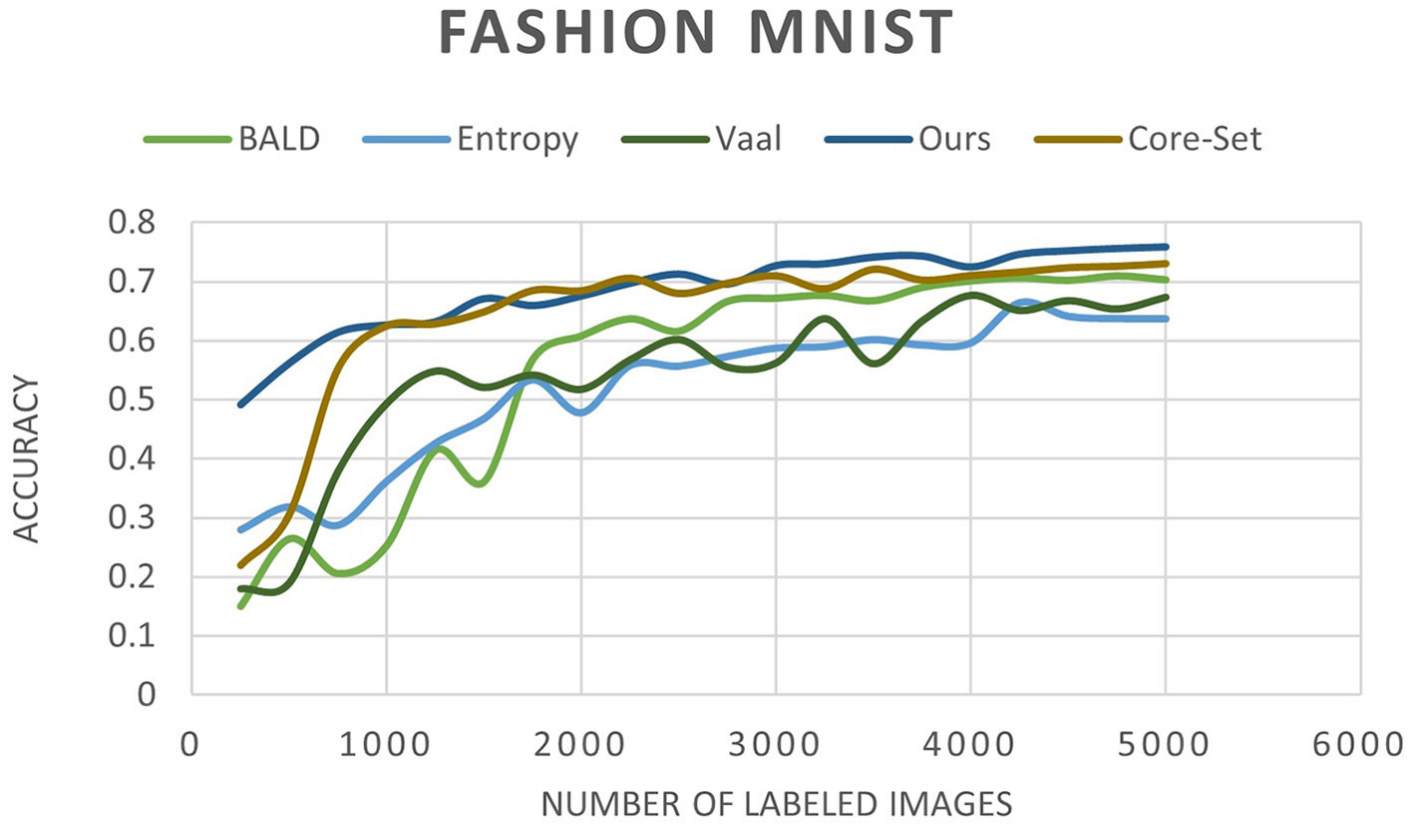
Accuracy of our method compared to other state-of-the-art methods as a function of the number of labeled images for the FashionMNIST dataset.

**Figure 11:**
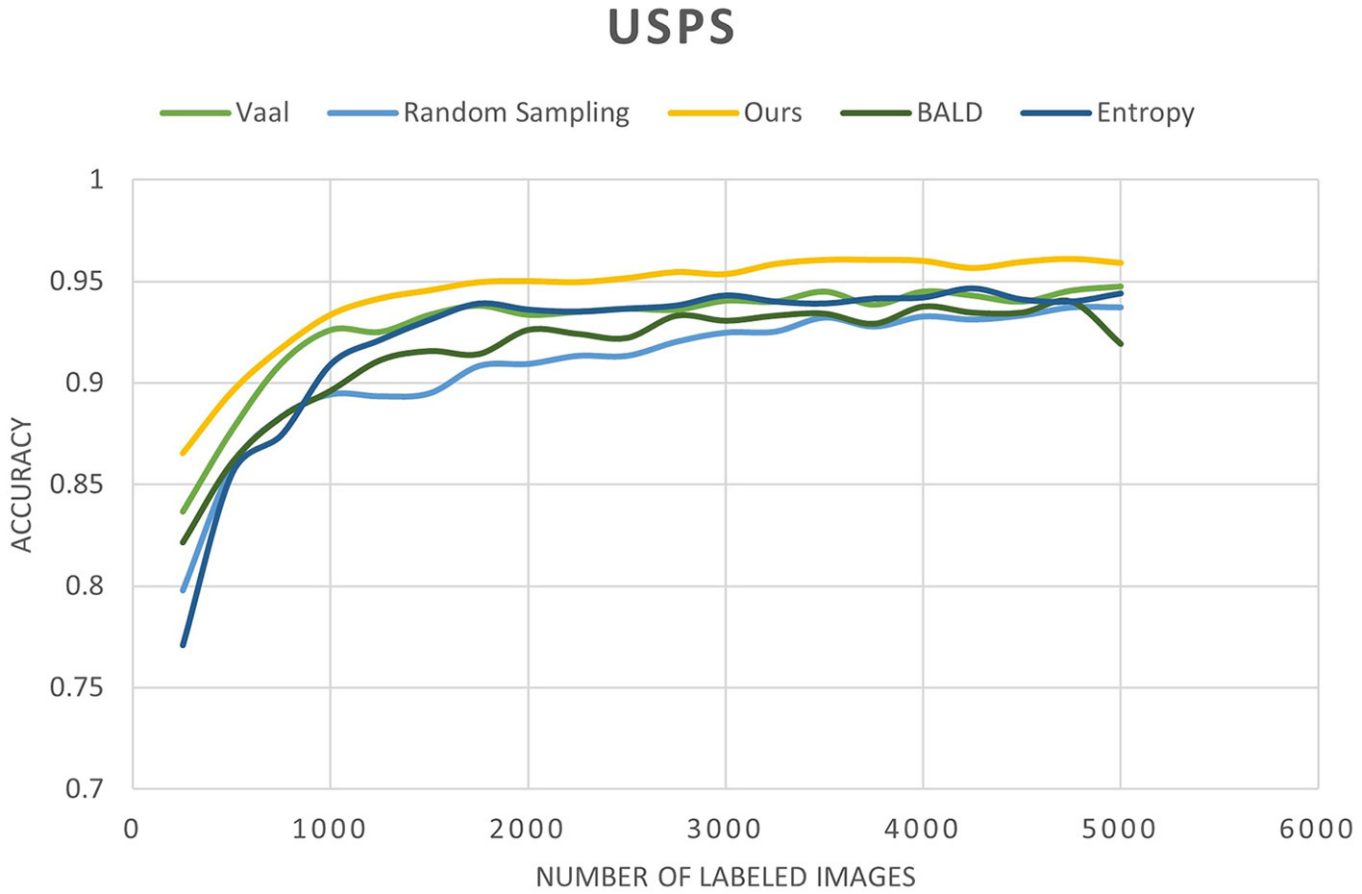
Accuracy of our method compared to other state-of-the-art methods as a function of the number of labeled images for the USPS dataset.

We conducted a comprehensive evaluation of our proposed method across multiple datasets, including MNIST, FashionMNIST, and USPS, as detailed in Tables 3–5. Our results showcase significant performance improvements over baseline methods, particularly evident in scenarios with limited labeled data. When compared to state-of-the-art techniques such as Core-Set Approach (Sener and Savarese, 2017), Variational Adversarial Active Learning (VAAL) (Sinha et al., 2019), and Bayesian Active Learning by Disagreement (BALD) (Houlsby et al., 2011), our approach consistently demonstrates competitive performance. Figures 9–11 showing our method comparing to the others (Notably, leveraging pre-trained) Notably, leveraging pre-trained clustering models contributes to achieving relatively high accuracy, particularly in scenarios with a scarcity of labeled samples.

**Table 3:** MNIST accuracy results on entropy sampling (Wang and Shang, 2014) BALD (Gal et al., 2017) Vaal (Sinha et al., 2019) Core-set (Sener and Savarese, 2017) and our method with 1, 3, 5, and 10% of the data labeled.

**Percentage of labeled data**	**Entropy**	**BALD**	**Vaal**	**Core-set**	**Ours**
1%	0.151	0.251	0.255	0.336	0.832
3%	0.600	0.701	0.735	0.805	0.910
5%	0.805	0.813	0.810	0.888	0.948
10%	0.935	0.945	0.917	0.928	0.983

**Table 4:** Fashion MNIST accuracy results on entropy sampling (Wang and Shang, 2014) BALD (Gal et al., 2017) Vaal (Sinha et al., 2019) Core-set (Sener and Savarese, 2017) and our method with 1, 3, 5, and 10% of the data labeled.

**Percentage of labeled data**	**Entropy**	**BALD**	**Vaal**	**Core-set**	**Ours**
1%	0.318	0.264	0.189	0.305	0.490
3%	0.468	0.360	0.520	0.627	0.671
5%	0.556	0.616	0.602	0.679	0.697
10%	0.637	0.703	0.673	0.729	0.758

**Table 5:** USPS accuracy results on entropy sampling (Wang and Shang, 2014) BALD (Gal et al., 2017) Vaal (Sinha et al., 2019) random sampling and our method with 3, 5, and 10% of the data labeled.

**Percentage of labeled data**	**Entropy**	**BALD**	**Vaal**	**Random sampling**	**Ours**
3%	0.770	0.821	0.836	0.797	0.865
5%	0.855	0.860	0.876	0.858	0.895
10%	0.909	0.896	0.926	0.894	0.933

### 4.6 Experiment analysis

**Figure 12:**
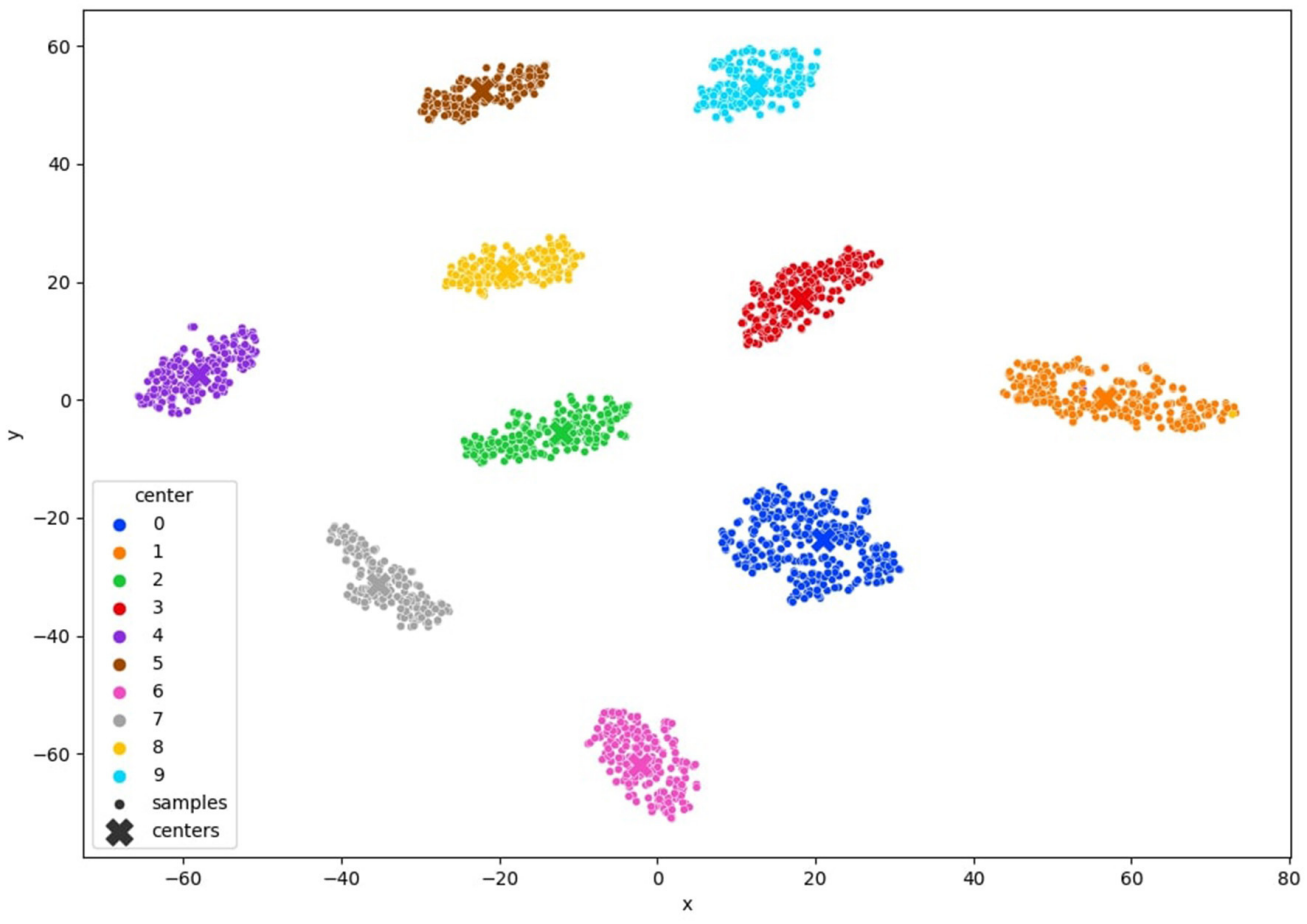
TSNE visualization of the clustered USPS latent space after convergence of our method with 10% of annotated samples.

To comprehensively validate the efficacy of our approach, we conducted an in-depth analysis of clustering quality throughout the training process. We monitored the evolution of clustering performance and visualized the t-SNE projections of learned latent space representations, as depicted in Figures 6, 7, 12. These visualizations offer insights into the structure of the learned representations, revealing distinct clusters corresponding to each class. The observed trends in clustering align well with the accuracy improvements reported in Tables 3–5, corroborating the effectiveness of our method.

In addition to accuracy comparisons, it's imperative to delve deeper into the performance metrics of our approach compared to baseline methods. For instance, on the MNIST dataset, our method achieves an accuracy of 91% with only 3% labeled data, outperforming the Core-Set Approach, which attains 80.5% accuracy. This notable performance gain underscores the superiority of our method in leveraging limited labeled data effectively.

## 5 Discussion

The integration of convolutional autoencoders, clustering, and a novel clustering contrastive loss in our semi-supervised active learning approach presents a unique and promising strategy for leveraging both labeled and unlabeled data in image classification tasks. By combining clustering with active learning, our method offers a distinctive approach that distinguishes it from previous methodologies.

A significant strength of our approach lies in its ability to extract valuable insights from unlabeled data by organizing it into clusters, thereby guiding the query selection process in active learning. However, the effectiveness of our method may depend on the quality of clustering initialization, which could potentially limit performance, particularly in scenarios involving complex, high-dimensional data. Exploring the applicability of our approach beyond image classification domains warrants further investigation.

Despite these potential limitations, our research represents a notable advancement in the realm of semi-supervised active learning. By integrating deep clustering, active learning, and contrastive learning principles, we address challenges associated with data scarcity, thereby enhancing model performance in resource-constrained settings. Moving forward, future research endeavors could explore the development of more robust clustering techniques, alternative representation learning methods, and synergistic combinations with other active learning strategies to further enhance performance and generalization capabilities.

Theoretically, the clustered representations derived by our approach hold promise for facilitating various downstream tasks, including data augmentation, domain adaptation, and the incorporation of weak or noisy labels. Such capabilities could prove invaluable in addressing the challenges posed by limited annotation scenarios. While our work contributes to the field, it also underscores the inherent challenges and opportunities associated with semi-supervised learning in real-world applications, paving the way for continued advancements and innovation in this domain.

It is essential to acknowledge the use of a smaller model architecture in our experiments. The complexity introduced by clustering necessitated the use of a smaller model to maintain tractability and computational efficiency. While this choice may have influenced our absolute performance metrics, it enabled us to explore the feasibility and efficacy of our approach within practical constraints. It is plausible that in subsequent studies, researchers may employ larger, more complex models to further improve performance.

## 6 Conclusions and future work

In this study, we have introduced a novel approach to image classification through a pool-based semi-supervised active learning technique. By integrating deep clustering and deep active learning, we aim to enhance classification accuracy by using fewer labeled images. Our method involves clustering feature vectors in the latent space that corresponds to images from *P*_*l*_ and *P*_*u*_, thereby obtaining a more informative representation of the latent space to support the active learning procedure. We have also incorporated a clustering contrastive loss to enhance the clustering of the latent space even with a limited number of labeled images. Cases where feature vectors in the latent space are not well grouped together or are far from their respective cluster centers are recognized as hard examples and are then queried for annotation by a human oracle.

Our empirical experiments demonstrated that our method achieves high classification accuracy even with a small number of annotations. The iterative combination of clustering with the suggested contrastive learning and query method leads to a more separated latent space, which in turn facilitates the classification process. Thanks to the clustering step, our method achieves high accuracy from the beginning. However, the clustering step may have a drawback for complicated datasets, as it can be challenging to cluster them effectively. We believe that future work can improve the clustering process to provide better clustering initialization even for complex datasets.

We used a convolutional autoencoder (CAE) to map samples to the latent space, but future work could explore more robust methods like a variational autoencoder that creates smoother and more connected latent spaces, which will help to improve clustering. Furthermore, our method is currently designed for image classification tasks, but it could be extended to other computer vision tasks such as semantic segmentation and object detection by inserting a suitable network head to the model for the requested task.

## Data availability statement

The original contributions presented in the study are included in the article/supplementary material, further inquiries can be directed to the corresponding author.

## Author contributions

HR: Conceptualization, Data curation, Investigation, Methodology, Resources, Software, Visualization, Writing—original draft, Writing—review & editing. AG: Conceptualization, Supervision, Validation, Writing—original draft, Writing—review & editing.
